# The Role of the Primary Care Transformation Lead: A Qualitative Case Study

**DOI:** 10.1177/21501319251327909

**Published:** 2025-03-16

**Authors:** Atharv Joshi, Judith B. Brown, Janet Dang, Jacobi Elliott, Shurabi Anphalagan, Shannon L. Sibbald

**Affiliations:** 1 Western University, London, ON, Canada; 2 Ontario Health, Toronto, ON, Canada; 3 St. Joseph’s Health Care London, London, ON, Canada

**Keywords:** integrated care, primary care, health systems, physician engagement

## Abstract

**Background:**

The introduction of Ontario Health Teams in Canada is a step toward achieving an equitable integrated system of care. The Middlesex-London Ontario Health Team (MLOHT) has been developed in parallel to the London-Middlesex Primary Care Alliance (LMPCA), a grassroots network for primary care physicians, health care administrators, and nurse practitioners. Key in the growth of the LMPCA was hiring a primary care transformation lead to support in engagement. This qualitative case study aims to describe the implementation of a primary care transformation lead within an integrated care setting through feedback from healthcare personnel.

**Methods and Findings:**

Family physicians, healthcare administrators, and administrative support personnel were recruited from the LMPCA and the MLOHT and interviewed. This analysis revealed 4 key components central to the role of a primary care transformation lead: (re)-building relationships, flexibility and adaptability, importance of role clarity, and motivation for change. Findings suggested that a primary care transformation lead can improve workflow among physicians by assisting in administrative tasks. Through streamlining information for primary care physicians, and building community networks, transformation leads can also enhance communication. Additionally, they can maintain an open environment for physicians to share their challenges to collaboratively develop solutions.

**Conclusion:**

This study exemplifies the role of primary care transformation leads in improving workflow, building networks, decreasing administrative burden, and facilitating an open environment in a primary care setting.

## Introduction

Primary care is the foundation of all health systems and serves a vital role in integrating care within a system.^1-3^ Primary care provides the initial point of contact services for patients as they move through the health care system.^4^ It involves multiple aspects of care, including diagnosis, treatment, illness prevention, health promotion, and the continuous management of patients with chronic conditions. Additionally, it acts as a vital entry point for individuals seeking care for acute or chronic illnesses and plays a key role in managing complex diseases.^4,5^ Primary care refers to health care services delivered to individuals (that can involve primary care providers). Primary health care refers to a broader approach toward health policy that not only focuses on primary care services at an individual level but also public health interventions at a population level.^4^ Canada has a publicly funded healthcare system that ensures Canadians have access to care across multiple sectors, including hospitals and community-based care.^6^ In Canada, provinces and territories such as Ontario hold primary jurisdiction over their health care, where they manage funding support, healthcare delivery, and uphold the federal Canada Health Act.^7^ Primary care is delivered through several different approaches (or models) in Ontario which is Canada’s most populous province. Over the past 20 years, the Ministry of Health and Long-Term Care (now the Ontario Ministry of Health and Ontario Ministry of Long-Term Care) has worked to reform Ontario’s healthcare system from a system that is hospital-centric to a system with an emphasis on patient point of care which allows for diagnostic tests to be performed by trained healthcare professionals closer to the patient, such as in outpatient settings, remote areas or community-based settings.^8^ To aid in this health care shift, Ontario is undergoing one of the largest health care system transformations with the introduction of Ontario Health Teams (OHTs).^9^ The introduction of the OHTs has demonstrated the government’s commitment to improving care through integrated care. As a policy intervention, OHTs are an integrated care model designed to improve care integration across sectors to advance an equity-oriented Quadruple Aim. The Quadruple Aim refers to improved health outcomes for people in communities, better patients, and health care provider experience, and provides better value per capita.^10,11^ OHTs will serve a critical role in improving health system coordination and enabling better team-based patient care catered to a community or geographical region.^12^ OHTs will include a range of sectors across the health care continuum ranging from primary care and mental health through to social services.^11^ OHTs are currently in various developmental stages with individual OHTs focusing on different priority populations such as chronic obstructive pulmonary disease, congestive heart failure, mental health and addictions and frailty. To date, there are 58 OHTs across the province with plans for developing new teams in the coming years.^8^

The Middlesex-London Ontario Health Team (MLOHT; previously known as Western Ontario Health Team) is in the geography of Middlesex County in Southwestern Ontario which is home to over 500,000 residents,^Click or tap here to enter text.11^ Within the region, the MLOHT and in parallel, a grassroots network of primary care physicians, health care administrators and nurse practitioners called the London-Middlesex Primary Care Alliance (LMPCA) has been developing. The LMPCA aims to represent the voice of primary care in Middlesex County and support primary care activities across the region. The LMPCA collaborated in formalizing the MLOHT and in developing governance structures to strengthen the local primary care sector.^13^ Key in the growth of the LMPCA was creating a position called the primary care transformation lead which was designed as a unique role in supporting family physicians and primary care organizations including project management, and organizing internal and external communication, to engaging professionals and care partners. While there is research supporting the value added with administrative support roles in terms of facilitating productivity,^14,15^ there are limited studies demonstrating a pragmatic example of this within primary care.

Lack of physician engagement is well documented as a current challenge within health system change and transformation.^16-18^ Physician engagement is a term that encompasses a physicians’ motivation to work to active contribution, commitment and involvement with the organization in which they work.^19^ Despite the benefits that are associated with physician engagement, many providers are unable to engage or take on leadership roles due to clinical commitments, teaching responsibilities and administrative duties.^14,20^ Currently, physicians in Ontario are required to do more administrative tasks than seen previously with estimates of roughly 40% of their clinical work (equivalent to 19 h) dedicated to administrative duties.^21^ Organizational policies can also be a barrier to physician engagement. The requirements of being adherent to health care and hospital protocols, guidelines, and regulations can create a reduction in autonomy, especially in circumstances when the physician voice may have been minimal or absent at the time of protocol or policy development.^20^

This research is part of a larger study that explores and describes the development of a regional primary care alliance (LMPCA) and the engagement of physicians in that process. The larger study highlighted the importance of physician engagement, with the transformation lead serving as a key catalyst in driving this effort. This role not only empowered physicians to excel in their clinical responsibilities but also encouraged their active participation in initiatives beyond patient care. This paper focuses on the role of the primary care transformation lead as an effective tool to increase engagement within primary care practitioners, and for this study, specifically primary care physicians. Our aim is to describe the development and implementation of a primary care transformation lead role within an integrated care setting (ie, Ontario Health Teams).

## Methods

The data reported in this paper are from a larger qualitative case study that focused on the development and implementation of a regional primary care alliance. The study received ethics approval from region’s Ethics Board prior to participant recruitment and data collection.

### Setting and Participants

The LMPCA, the primary focus of this study, is located within the Southwestern Ontario region, with several health service organizations, academic teaching hospitals, and a university and college, which have made it an important hub for health care innovation and education partnerships. The LMPCA is a grassroots and volunteer-based organization that focuses on representing the values, vision, goals and voice of primary care within the London-Middlesex region. The network consists of primary care physicians, health care administrators, nurse practitioners and support personnel (such as a communications lead and transformation lead). The LMPCA is a separate entity, with roots from its predecessor, the Southwest Primary Care Alliance founded in 2017.

We applied the Consolidated Criteria for Reporting Qualitative Research (COREQ; Supplemental Data) framework to enhance the quality of reporting. This framework guided our methods to establish an organized approach to data collection and analysis, strengthening the reliability of our findings.

Participants were recruited from both the LMPCA and the MLOHT and consisted of 3 main groups of participants: (1) family physicians; (2) health care administrators; and (3) administrative support personnel. Recruitment was conducted through convenience sampling from both organizations through broad approaches such as emails, webinars, Townhalls and social media. The participants were from the broader study regarding physician engagement.

### Data Collection

Consistent with case study methodology, multiple data sources were used for data collection.^22^ Data was collected through key informant interviews, document analysis, and an environmental scan. All participants received a full explanation of the potential risks and benefits associated with the research project as well as a Letter of Information prior to collecting written informed consent.

First, interviews were conducted with individuals who were part of the Executive Council of the LMPCA, Operations Team or Coordinating Council of the MLOHT, or engaged with primary care in some capacity. Interviews, conducted virtually through the videoconferencing platform Zoom, lasted 45 min and were transcribed verbatim through the Transcript Heroes Transcription Services (Toronto, ON, Canada). The document analysis was used to explore the major events of the LMPCA, to outline the development of the LMPCA and primary care engagement activities within the Middlesex-London region. This involved systematically examining and interpreting documents or other forms of communication (eg, PowerPoint presentations, letters, reports, policy documents).

An environmental scan was used to identify similar grassroots primary care organizations in Ontario and to assess/analyze whether they were similar to the LMPCA. The factors considered when conducting the environmental scan were whether the primary care organization was self-governing or formalized by Ontario’s Ministry of Health. Another filter of the environmental scan also looked at whether the involvement of physicians was voluntary and whether they assumed roles similar to that of the transformation lead whether it used a grassroots approach. Lastly, it was seen that the primary care organization had a partnership with their region’s OHT.

### Data Analysis

The interview data was analyzed using inductive content analysis supported by NVivo 13.^23^ Coding began with data immersion and looking for common concepts and ideas.^23^ All transcripts were analyzed by 1 researcher (A.J.), with the rest of the research team reviewing approximately 50% of the data. Our analysis was triangulated with multiple data sources (field notes and document analysis) as well as through regular team meetings with the entire research team.^23^ Themes were created through discussion with the research team and the iterative and constant comparison within and across all data sources. Document analysis was incorporated at this stage to support and validate our findings and ensure congruence of themes. For this study, a third round of purposeful, or targeted coding was done specifically looking across all data sources for information around the primary care transformation lead.^24^ These multiple methods worked together to help us develop a rich and comprehensive understanding and description of the role of the primary care transformation lead in the development of the LMPCA.

During the research process, researchers generated written reflexive notes to incorporate the interviewer’s thoughts and assumptions into data analysis and interpretation.^25^ Member checking was done after all preliminary data analysis was done, involving participants from LMPCA and MLOHT. In this member checking phase, a summary of the study and its themes was disseminated via email to all 13 participants for review. Following the review of the summary, 80% of the participants provided feedback to elucidate or endorse the study’s findings.

## Results

Overall, 13 participants participated in the study which included family physicians, health care administrators and support personnel from the LMPCA and the MLOHT (Table 1).

**Table 1.** Demographics and Characteristics of the Study Participants (n = 13).

Participant characteristics	N (%)
Age (years)	
30-39	4 (31)
40-49	5 (39)
50-59	2 (15)
60-69	2 (15)
Gender	
Male	5 (39)
Female	8 (61)
Participant category	
Family Physician	4 (31)
Health Care Administrator	3 (23)
Administrative Support Personnel	6 (46)
Total	13

Overwhelmingly, when participants were asked to talk about the development of the LMPCA, they all pointed to the role of the primary care transformation lead as a key success. A physician pointed out that “the most critical piece to the LMPCA was having [a transformation lead] who did enormous groundwork and helped be that project planner” (Physician 2, Interview).

Given the importance attributed to this role, we wanted to learn more about its specifics and central components. Our analysis revealed 4 key components central to the role: (re)-building relationships, flexibility and adaptability, importance of role clarity, and motivation for change. We will explain each of these components in turn after a brief description of the LMPCA and the primary care transformation lead role.

### Brief Description of LMPCA

LMPCA has about 20 members across their Executive Council with many physicians, nurse practitioners, health care administrators and other crucial stakeholders who have enrolled in the communications and activities of the LMPCA. The organization holds in-person and virtual town halls open to the public, physicians, and primary care providers multiple times a year focused on current issues in primary care. LMPCA also sends out monthly newsletters and communication e-Blasts providing information on ongoing activities of the LMPCA and how physicians and other interested members are currently engaged. Participants in our research described how the LMPCA took pride in having the region’s first primary care transformation lead—most participants believed this was unique across the province stating that “we’ve been able to hire the region’s very first [transformation lead] position and it’s the [transformation lead] position that has been one of the catalysts in engagement” (Physician 1, Interview). The LMPCA has demonstrated notable success and progress in addressing the need for a coordinated approach within the sector. For example, through the Personal Protective Hub initiative which included organization of vaccination clinics, and distribution of equipment (personal protective equipment [PPE]) during the COVID-19 pandemic. Also, through supporting the work of the OHT within the London-Middlesex region. The primary care transformation lead played a major role in ensuring the above activities were successful throughout the region.

### Description of a Primary Care Transformation Lead

Documents (eg, job advertisements) described the role of the primary care transformation lead as an independent and dynamic role that supports physicians, nurse practitioners and health care administrators as well as the patients and the community (LMPCA Job Posting, Document 2022). The primary care transformation lead had responsibilities to oversee and facilitate systems to improve the quality, efficiency, and effectiveness of healthcare delivery. This role involved working with healthcare providers, stakeholders, and communities to identify and implement evidence-based practices and strategies promoting patient-centered care, improved outcomes, and cost-effective use of resources.

The position was funded through 5 separate primary care entities within the region, 3 family health teams, 1 Nurse Practitioner-led clinic, and 1 community health center. Leadership within these groups agreed that in order to move forward as a sector, there needed to be a solid infrastructure (information supported through interviews and document analysis).

If you’re trying to build a sector you need an infrastructure and [the transformation lead] brought that infrastructure for us . . . I keep saying it’s not rocket science to think that if we want to have a tight sector, we need good strong administrative leadership to work with us and to bring us all together and to be the conductor of the orchestra. (Physician 3, Interview)

Within the LMPCA, when introduced, the primary care transformation lead did not have predefined duties, allowing the role to evolve organically based on emerging needs and opportunities. This flexibility enabled the transformation lead to adapt as the broader context within which they worked developed and changed. The first key role was understanding and supporting providers’ needs. This involved a hands-on approach to understanding the unique challenges and opportunities within each clinic. The transformation lead scheduled meetings both before and after clinic hours to minimize disruption to patient care while ensuring meaningful engagement. The transformation lead also supported physicians in their leadership development by supporting providers with skills to navigate system-level changes.

### (Re)-building Relationships

The transformation lead played an important role in establishing a strong foundation of leadership within primary care through the building and re-building of relationships. In London-Middlesex, there has been a history of poor relationships and lack of trust amongst primary care physicians, health care administrators, and organizations. The transformation lead addressed these challenges through proactively building strong relationships with family physicians in the community by going door-to-door and getting to know the clinicians on a personal level. Participants highlighted the effectiveness of the role’s design which prioritize relationship building in primary care.

We got very fortunate with [the transformation lead] because there was a bit of an X factor in [their] natural skill set, building networks, communicating. [They] connected with physicians and build a relationship at a personal level as well as had tremendous vision. I think personally, that’s what gained the traction where [they] helped move past the previous hesitancies and bring a degree of energy to support primary care. (Physician 4, Interview)

The transformation lead’s leadership helped empower physicians to be engaged in issues and concerns central to primary care. A key example of this was leading the co-creation of a shared vision for the team and removing any administrative barriers that came in the way of physicians. The transformation leads role of team-engagement removed the need for physicians to advocate for “nonsensical issues . . . it was really about building a natural dyad with us physicians” (Physician 4, Interview). This allowed physicians to prioritize in their clinical activities and continue with their involvement in primary care work. The systemic approach to relationship building facilitated by the transformation lead role, created a foundation to sustained collaboration and engagement in primary care.

### Flexible and Adaptable

As mentioned above, there were limited parameters around the transformation lead activities, this meant the role (by design) was flexible and required them to adapt to local challenges and conditions. While there were no fixed expectations for the primary care transformation lead, being flexible and adaptable was crucial to effectively respond to the needs of the LMPCA. A key example of this was during the COVID-19 pandemic. Unexpected events during the pandemic required an adaptable and flexible approach to effectively manage crises. The transformation lead centralized information and share updates in a streamlined way. Participants experienced this during the COVID-19 pandemic when there was a shortage of PPE across the region.

With the COVID-19 pandemic there was a lot of things [happening] very quickly and there was lots of information to be shared and we found that the LMPCA and [the transformation lead]’s skills were able to play a role in that so there was a centralized place to collect PPE and get PPE out to our colleagues that needed it, so that was a big help. (Physician 3, Interview)

This was seen when the primary care transformation lead adapted their approach to accommodate physicians’ busy clinical schedules.

“I’m going to have a meeting tonight from six to eight o’clock.” But [the transformation lead] rearranged [their] life because [they] understood how we [physicians] work. And that’s why someone like [the transformation lead] is so rare. Because how do you find someone who’s willing to be that flexible and adaptable and smart and intuitive to match up? (Physician 4, Interview)

Physicians noted their appreciation for the primary care transformation lead’s role flexibility in delegating tasks and empowering team members. The transformation lead provided autonomy to team members to make decisions, enhancing their sense of ownership and job satisfaction. The flexibility and adaptability of the meetings helped ensure the sustainability and the momentum of the primary care related activities in the LMPCA.

Doctors don’t have a lot of time, so you need to do this work between like 6:30 a.m. and 9 a.m., or like 7 p.m. to 10 p.m., so outside of traditional hours that many of us work. So I think [the transformation lead]’s role recognized that it’s going to happen at odd hours and still supported the work and empowered those physicians. (Health Care Administrator 2, Interview)

The transformation lead coordinated with the schedules of physicians, enabling them to contribute effectively despite their individual work constraints.

### Importance of Role Clarity

The third component critical to the success of the transformation lead was ensuring role clarity. Many participants highlighted that the transformation lead supported physicians from a non-clinical standpoint. This duality combining administrative expertise with an understanding of clinical realities was pivotal in promoting the success of the LMPCA. However, the role was not merely defined by this duality but also by the clarity of its scope, which allowed the transformation lead to navigate the complexities of primary care transformation effectively. One of the primary responsibilities of the transformation lead was to remove anticipated burdens or challenges associated with physician engagement initiatives. This included addressing logistical, administrative, and organizational obstacles that often hindered effective participation by physicians. By focusing on alleviating these barriers, the transformation lead enabled physicians to concentrate on delivering high-quality care. Physicians greatly recognized the significance of this duality and the role’s well-defined scope. As one participant noted:

The duality of leadership in healthcare, if it works, is so powerful. And that’s what [the transformation lead] brought. [They] did not try to be a clinician. [They] learned about what primary care was by meeting many physicians’ concerns and requests about what their unmet needs are. What would make it better, what would make it easier to deliver high-quality care? (Physician 2, Interview)

The transformation lead’s role was distinct in its responsibilities. Including understanding and addressing physician concerns, project planning, boundary setting and boundary spanning. This role clarity not only supported physicians but also ensured the success of the LMPCA by aligning administrative efforts with clinical priorities. The ability to navigate both administrative and clinical perspectives without conflating the 2 fostered trust and collaboration among stakeholders.

### Motivation for Change

The last part of the successful transformation lead for the LMPCA was having an individual motivated to bring change to the sector. The main duties of the transformation lead encompassed managing administrative tasks in primary care, championing and assisting physicians, and adeptly managing complex situations they were confronting. While there were other operational support personnel within the organization, their roles were primarily focused on addressing individual clinical or logistical needs rather than engaging with physicians to build relationships or to be engaged in system reform. The transformation lead’s role was distinct, focusing on fostering physician engagement, understanding unmet needs, and driving system-wide change. Many individuals acknowledged this role as ideal for those with a fervor for primary care and a drive to push for change within the sector.

A lot of times it’s an admin focus that you’ve just got to basically keep the lights on, get everything going . . . But the other half of it where you can kind of design it and really push for meaningful change in the healthcare system and make sure that there is that voice for primary care at all different levels. It is a very effective position to have. (Health Care Administrator 3, Interview)

Many people noticed that this motivation led to different roles and viewpoints, helping to understand the next improvements needed in the local primary care sector. This included introducing new ideas and projects strategically which included the PPE hub as mentioned above and “building individual relationships with the physicians to understand their concerns” (Physician 2, Interview).

## Discussion (Lessons Learned)

This study aimed to describe the development and implementation of a primary care transformation lead role in an integrated care setting such as an OHT. The LMPCA was chosen because it included highly engaged participants who organized and pushed toward addressing the need for a coordinated approach within the sector. Member checking was conducted with the original participants (consistent with qualitative methodology) to ensure that the findings accurately represented their experiences and perspectives. Member checking enhances the credibility of the findings by directly validating them with participants involved.

We found 4 key components supporting the unique and effective role of the primary care transformation lead: rebuilding relationships, flexibility and adaptability, importance of role clarity, and motivation for change. Through exploring the LMPCA and the role of the transformation lead, we saw how physicians and administrative personnel could work in duality, providing effective leadership within a regional organization.

A key barrier to the physician’s engagement and workplace efficiency occurs in the mental stress and burnout they face with increasing administrative burden.^26^ Administrative burdens in healthcare for physicians in Canada refer to the various non-clinical tasks and paperwork that doctors are required to complete as part of their practice. These tasks can include billing and coding procedures, prior authorization requirements, documentation for insurance claims, regulatory compliance, filling out forms for government programs, and managing electronic health records.^21^ This administrative burden not only limits a physician’s full potential but also impacts and detracts the time spent engaging with patients or health systems work.^27^ Before the hiring of the LMPCA transformation lead, it was the physicians who had scheduled meetings and organized LMPCA activities as mentioned in the results. The transformation lead alleviated the administrative burdens of LMPCA executives, distinct from clinical administrative tasks (clinical administrative work was still conducted by the physicians themselves). This streamlined processes, allowing physicians to focus on key leadership activities and health systems work alongside their clinical duties, without adding to their workload. Research on administrative burden in Canada is currently limited, but Lavergne et al^28^ have developed a study and protocol to explore this issue. They are examining ways to address concerns and develop new strategies to make this efficient for family physicians as well as nurse practitioners in Nova Scotia. While their study focuses on clinical administrative burdens, it shares similarities in that they are trying to devise a plan to make administrative tasks more efficient and effective. By hiring the primary care transformation lead, workflow and efficiency among physicians improved as they were supported in organizational administrative tasks related to LMPCA. By streamlining information for physicians with busy schedules, transformation leads offered efficient and effective approaches toward communication in primary care. The transformation lead role complemented traditional operational models by bridging clinical and administrative priorities. Acting as a liaison, the transformation lead ensured that system reform initiatives were informed by clinical realities while aligning with organizational objectives, addressing critical gaps not covered by standard business management roles.

Another crucial aspect was someone who was driven and interested in making a difference. By maintaining close contact, the primary care transformation lead created an open environment for physicians to share their perspectives, challenges, and through combined efforts, determine evidence-based solutions. This ensured that the physicians could focus strictly on their clinical duties and the transformation lead focused on helping the physicians do their jobs without any obstacles in the way. Evidence of this is seen through participants who highlighted that the duality of the support received by the transformation lead promoted the success of the LMPCA. Having an individual to support physicians in navigating their obstacles can also ensure increased satisfaction and morale in a healthcare team.^29^

The primary care transformation lead also kept a flexible and adaptable schedule which allowed them to work with the uncertain schedules of physicians. Understanding the unpredictable and unconventional schedule of primary care physicians, a primary care transformation lead can ensure close communication with physicians by maintaining a flexible schedule. In a study of primary health care teams, Brown et al^30^ found that scheduled team meetings were vital for communication as they provide a safe space for individuals to participate in problem solving collaboratively. Understanding the importance of team meetings in primary health care teams, primary care transformation leads can maintain open communication by scheduling such meetings with physicians. The flexibility of the primary care transformation lead provides space for team members to have autonomy, which is critical in situations where physicians must schedule their primary care-related activities around their clinical duties. Creating a role with flexibility and adaptability at its core can be valuable in certain contexts—especially in system reform. However, it is important to clarify that this approach is not about having no expectations but rather focusing on role fluidity tailored to the specific needs of the organization or situation. In practice, this means conducting an environmental scan to identify the key priorities and gaps in that context. The individual in the role would then work proactively to address these priorities, leveraging skills like relationship-building, problem-solving, and adaptability. This approach can foster innovation and responsiveness in dynamic environments.

One key finding of the primary care transformation lead was how they helped build networks among physicians, improving communication within the primary care sector. This was evident during the pandemic when the transformation lead had created The Personal Protective Equipment hub. The transformation lead worked with physicians and organizations across the Middlesex-London region helping physicians connect and get PPE due to the shortage that occurred across the region. Participants highlight the advantage of having a transformation lead who provided a structure for collaboration during a time of a pandemic. This can apply broadly to increase knowledge about community services among physicians. In a study by Kiran et al,^31^ it was found that physicians had relatively low awareness of centralized intake services in their region, such as a point of contact to navigate community services. Reasons for this low awareness could be attributed to lack of advertisement of such services, or physician’s need to focus on changes to medicine rather than community services.^31^ Understanding this current gap, a primary care transformation lead can ensure physicians have improved knowledge of community services, enhancing patient’s access and overall community wellbeing. Within the community of physicians and health leaders, it was widely recognized that this role excelled in fostering strong connections and relationships. The role was mostly building relationships and alleviating any burdens for physicians so they can focus on what they do. While not easily measurable, this impact was clearly reflected in interviews and acknowledged by healthcare leaders in the London-Middlesex region.

Our findings are specific to the LMPCA’s context and reflect a rich, contextualized understanding of the transformation lead’s role in supporting primary care physicians within this system. These insights can serve as a foundation for adaptation and inform similar initiatives in other settings with analogous challenges.

## Strengths and Limitations

A strength of this study was the group of participants from diverse occupational backgrounds within the clinical setting, including physicians, healthcare administrators, and administrative support personnel. These participants were highly involved in the development and implementation of the LMPCA and offered many unique perspectives toward the implementation of a primary care transformation lead including insight into the benefits of employing a transformation lead. A limitation of the study includes the timeline for recruitment of participants which commenced in late fall, as it occurred shortly after the waves of the COVID-19 pandemic and could contribute to participants availability to be interviewed. Alongside this, the timeline also conflicted with the season of respiratory viruses such as influenza, causing a greater influx of patients and increased physician workload. Since convenience sampling was applied, there is potential that those most interested in the program were more likely to participate and provide positive feedback. Furthermore, since the study primarily focused on the LMPCA and OHTs, and even more specifically primary care physicians, the lessons learned may not directly be transferable to other contexts within primary care. We recognize that the absence of a theoretical framework limits the ability to draw explicit causal links between the transformation lead interventions and the observed outcomes. Future research should integrate such frameworks to better understand the pathways to systems change. While our findings suggest promising impacts, future studies might benefit from longitudinal designs to evaluate the sustainability of outcomes and to explore the mechanisms driving systems change in greater depth.

## Conclusion

This study focuses on the development and implementation of a primary care transformation lead role within an integrated care setting, aiming to enhance primary care engagement. This study is part of a broader exploration into the development of a regional primary care alliance (LMPCA) and the engagement of physicians within it. Specifically, it delves into the role of the primary care transformation lead as a strategy to enhance primary care engagement within a regional organization (the LMPCA) and Ontario Health Teams, an integrated care setting. By understanding the implementation of this role, this research contributes to understanding effective approaches to enhancing primary engagement in healthcare transformation efforts. The research also highlights the importance of innovative leadership roles and structures in overcoming barriers and facilitating meaningful physician involvement in shaping the future of healthcare delivery.

## Supplemental Material

sj-pdf-1-jpc-10.1177_21501319251327909 – Supplemental material for The Role of the Primary Care Transformation Lead: A Qualitative Case Study Supplemental material, sj-pdf-1-jpc-10.1177_21501319251327909 for The Role of the Primary Care Transformation Lead: A Qualitative Case Study by Atharv Joshi, Judith B. Brown, Janet Dang, Jacobi Elliott, Shurabi Anphalagan and Shannon L. Sibbald in Journal of Primary Care & Community Health
